# Cybersecurity in the financial sector and the quantum-safe cryptography transition: in search of a precautionary approach in the EU Digital Operational Resilience Act framework

**DOI:** 10.1365/s43439-025-00135-7

**Published:** 2025-03-05

**Authors:** Laima Jančiūtė

**Affiliations:** Institute for Logic, Language and Computation, Faculty of Science, University of Amsterdam, Amsterdam, The Netherlands

**Keywords:** DORA, Financial cybersecurity, Quantum threat, Precautionary principle, Post-quantum cryptography

## Abstract

An ever more digitalised financial sector is exposed to a growing number of cyberattacks. Given the criticality and interconnectedness of this sector, cyber threats here represent not only operational risks, but also systemic risks. In the long run, the emerging cyber risks include developments in quantum computing threatening widely used encryption safeguarding digital networks. Globally in the financial sector, some initiatives have already been taking place to explore the possible mitigating measures. This paper argues that for an industry-wide transition to quantum-safe cryptography the precautionary principle is relevant. In the EU, financial entities now have to be compliant with the Digital Operational Resilience Act strengthening ICT security requirements. This research traces the obligation to adopt quantum-resistant precautionary measures under its framework.

## Introduction

The financial sector is part of critical infrastructure, i.e. it is one of the sectors that are vital for the effective functioning of societies. “The financial system performs a number of key economic functions which support the real economy” [19, p. 7] In recent decades, the global financial system has become increasingly digitalised, interconnected and interdependent and “has come to rely critically on robust information and communications technology … infrastructures” (Ibid, p. 2). As a consequence, financial entities and infrastructures have become a frequent target of cyberattacks which have been growing exponentially (see e.g. [27]) and have been becoming ever more sophisticated and coordinated, potentially constituting not only operational risks, but also systemic risks threatening wider financial stability if trust in the financial system is compromised as a result of a cyber incident [19].

“‘Systemic risk’ means a risk of disruption in the financial system with the potential to have serious negative consequences for the internal market and the real economy. Financial stability in general refers to the proper functioning of financial markets in support of the real economy, i.e. their capacity to absorb shocks, continue providing the key economic functions … Financial stability is threatened when shocks cannot be absorbed and amplifying dynamics such as bank runs, liquidity and lending freezes, fire sales, market crashes or hyperinflation occur” (Ibid, p. 22; see also [25]).

Concerns by authoritative sources such as the European Central Bank chief Christine Lagarde have been voiced that the next financial crisis may be triggered by a cyberattack [43]. Alongside the enduring cyber threats, there are new emerging challenges, among which the imminent advent of cryptographically relevant quantum computers capable of breaking most of the currently used encryption underpinning the security of digital networks ([31] see also [7] and [41]). “There is no doubt that quantum computing represents a major risk to financial stability” [4, p. 17]. To mitigate these risks, novel encryption methods are being developed capable of withstanding quantum-enabled attacks. It is necessary for financial institutions to adopt these new methods so that “critical aspects of financial operations such as data security and communication privacy” are not undermined [44, p. 22]. Financial entities “should develop plans to migrate current cryptography to quantum-resistant algorithms” [11, p. 5]. Quantum computers pose a cybersecurity threat already today, because information can be intercepted now and retroactively decrypted later and because transitioning to new cryptographic standards is likely to take decades [24]. This is further compounded by the long-term sensitivity of financial data [4, p. 2, 6, 10, 17].

Given the criticality of this sector, the financial sector has been a highly regulated domain including its cybersecurity aspects ([15, 28] see also [14]). To introduce a comprehensive and harmonised framework strengthening the ICT security of a range of European financial entities, the EU institutions adopted in late 2022 the Digital Operational Resilience Act (DORA) (Regulation (EU) 2022/2554 [39])—a sector-specific legislation that applies from 17 January 2025. This article explores whether its provisions provide basis for financial entities to adopt quantum-safe cryptographic techniques and whether the approach taken in this piece of legislation can be aligned with the precautionary principle. Section 2 sets the scene by outlining cryptographic threats in the financial sector and the measures that some financial institutions have been taking to mitigate those threats. Section 3 discusses the relevance of the precautionary principle in the digital environment. Section 4 analyses the DORA framework with regard to quantum-safe cryptography transition and the precautionary approach. Section 5 concludes.

## Setting the scene

Encryption is one of the elements used to secure communication [36, p. 226]. The development of quantum computers threatens financial stability as it may render asymmetric (public-key) encryption, which the financial sector heavily relies on, obsolete. Potential attacks by quantum computers represent a number of cryptographic risks specific to the financial industry, such as

- “the security of stored personally identifiable information”,
- “authentication vulnerabilities in wholesale payment systems”,
- “compromise of inter-bank system interfaces”,
- “compromise of distributed ledger technology based financial instruments”,
- “authentication vulnerabilities for privileged access to infrastructure and systems”,
- “authentication vulnerabilities in consumer payment systems”,
- “vulnerabilities in software and system integrity”,
- “altered financial transaction records”—the private ledger and the public ledger [44, p. 23–28].

In sum, successful attacks against currently used algorithms “would compromise connections used by the financial system, including mobile banking, e‑commerce, payment transactions, ATM cash withdrawals, and VPN communications, to name just a few” [41].

The main method in protecting systems from the quantum threat is the adoption of post-quantum cryptography (PQC)—a set of cryptographic algorithms that are meant to be able to shield from quantum-enabled attacks. Since late 2016, a central effort in developing and standardising these algorithms has been taking place under the auspices of the US National Institute of Standards and Technology (NIST) [32]. In July 2022, the NIST announced the first four selected quantum-resistant cryptographic algorithms to be standardised: one for general encryption (CRYSTALS-Kyber) and three for digital signatures (CRYSTALS-Dilithium, FALCON and SPHINCS+) [33]. In summer 2023, NIST published the draft standards for public comments for three of them [34]. In August 2024, NIST finalised these first three standards “encouraging computer system administrators to begin transitioning to the new standards as soon as possible” [35].

At the same time, various initiatives have been taking place in anticipation of the quantum-safe transition in the financial sector. The Bank of France has successfully trialled PQC data exchange security solutions [2]. Subsequently, a joint experiment (Project Leap) has been conducted by the Bank of France, the Deutsche Bundesbank and the Bank for International Settlements’ Innovation Hub Eurosystem Centre, aimed “at quantum-proofing the financial system, starting with central bank processes” and “raising awareness among the central banking community” [4, p. 2]. Its first phase tested “the implementation of post-quantum cryptographic protocols to central bank use cases such as payments. A quantum-safe environment was created to secure infrastructures against the interception of data in transit” (Ibid). In Project Leap, “all the algorithms selected by NIST for standardisation were tested” as well as the FrodoKEM algorithm recommended by the French and German cybersecurity authorities (Ibid: p. 13, 20). Besides, in 2024, the Bank of France and the Monetary Authority of Singapore announced that they successfully completed a joint experiment in PQC “conducted across continents over conventional Internet technologies” trialling the use of PQC algorithms for the encryption and signing of emails [3].

The Bank of Canada has published a working paper on privacy-preserving post-quantum credentials for digital payments [26]. The Bank of Italy published a paper on quantum-safe payment systems focussing in particular on quantum random number generation and quantum key distribution technologies [6]. The Bank of Italy also published another paper on the implications of the quantum challenge and strategies for a secure financial system [1]. The central bank of Brazil together with partners conducted a feasibility study of the application of PQC methods to the Brazilian instant payment system [21]. Reportedly, some operators of the global financial industry have also already been preparing for potential quantum attacks [9, 22, 23]. In addition, in 2024, a dedicated Quantum Safe Financial Forum was created by Europol’s European Cybercrime Centre (EC3), in close cooperation with the EC3 Advisory Group on Financial Services. Among its objectives are driving a coordinated approach to the transition to PQC in the financial sector and discussing potential solutions for this sector [20]. As can be seen, precautionary measures against the quantum threat are being explored within the financial industry. The next section discusses the relevance of a precautionary approach.

## The need for a precautionary approach

The often-cited definition of the precautionary principle is provided as Principle 15 in the 1992 UN Rio Declaration on Environment and Development, which states that

“In order to protect the environment, the precautionary approach shall be widely applied by States according to their capabilities. Where there are threats of serious or irreversible damage, lack of full scientific certainty shall not be used as a reason for postponing cost-effective measures to prevent environmental degradation” [45].

The precautionary principle aims to enable decision-makers to act more promptly (to adopt precautionary measures) in situations where scientific evidence about hazards is uncertain or inconclusive and the stakes are high [5]. In the EU, the precautionary principle is enshrined in Article 191(2) of the Treaty on the Functioning of the EU. It emerged as a principle of environmental policy, but has since been extended to other areas and is now recognised as a general principle of EU law “which should in particular be taken into consideration in the fields of environmental protection and human, animal and plant health” [17, p. 9]. The precautionary principle is of particular relevance to the management of risk (Ibid, p. 2).

“Recourse to the precautionary principle presupposes that potentially dangerous effects deriving from a phenomenon, product or process have been identified, and that scientific evaluation does not allow the risk to be determined with sufficient certainty” (Ibid, p. 3).

The precautionary principle can thus be invoked to address both perceived and real risks [47, p. 2]. It has been becoming ever more significant in the context of the so-called “information society” [42] and digitalisation [38].

“Digital technologies and services are linked to complex, uncertain, and ambiguous impacts and consequences of their development, application, and use, and cause multiple ripples effects throughout many crucial systems of society. Potential threats that can be characterized as systemic risks include risks of failure, cybercrime, cybersecurity, misuse of data, protection of privacy, inequitable access to digital services, and many others” [40, p. 1911].

“The digital world offers a complex of dependencies similar to nature—small disturbances at one point could have major and irreversible consequences elsewhere” [37, p. 52]. Digitalised societies will be confronted with the same kind of issues “that were earlier relevant concerning genetically modified organisms and chemicals” [38, p. 3]. As the impact of digital technologies is increasing, “there is a growing need to apply the precautionary approach, especially to technologies exempted from standard risk governance frameworks” (Ibid, p. 21). In the case of the quantum threat to cybersecurity, a precautionary response encompassing migration to PQC is justified since the consequences of a false negative would be unconditionally unacceptable [48]. Also, overall, it is reasonable to consider the application of the precautionary approach in cases of systemic risks “due to high levels of complexity and uncertainty” [40, p. 1907].

“In the context of financial stability and cybersecurity, the precautionary principle requires … action already when there is a *risk* to the stability of the financial system. The mere possibility of damage is enough; a concrete probability is not required” ([8, p. 1156], original emphasis).

The next section analyses whether DORA—a landmark piece of legislation in the field of financial cybersecurity—can be aligned with the precautionary principle.

## DORA and the quantum-safe cryptography transition in the financial sector

A whitepaper on quantum security for the financial sector by the World Economic Forum and the UK Financial Conduct Authority suggests that regulators should primarily clarify how existing “frameworks apply to the quantum threat and cryptographic management more broadly” [46, p. 9]. “Where there is concrete evidence of gaps in existing frameworks, new regulation should be developed” (Ibid). A new EU-wide law—DORA—has recently been introduced that addresses cybersecurity in the financial sector. One of its post-legislative acts pays a close attention to cryptographic governance, as discussed below.

DORA was proposed in September 2020 and adopted in December 2022 as part of the EU Digital Finance Package. DORA is a cross-sectoral regulation and applies to 21 different types of financial entities, thus bringing harmonisation of the rules pertaining to operational resilience for the financial sector. It covers such key areas as ICT risk management, ICT incident management and reporting, testing of the operational resilience of ICT systems and the management of ICT third-party risks with the aim of preventing and mitigating cyber threats [16, p. 1, 5]. Besides, it acts as *lex specialis* to the Network and Information Security 2 Directive (Directive (EU) 2022/2555 [12]) and to Article 11 and Chapters III, IV and VI of the Directive on the Resilience of Critical Entities (Directive (EU) 2022/2557 [13]) [16 p. 1].

DORA recognises the systemic nature of cyber risks [29] referred to in multiple recitals and articles. Some of its parts lay down rather prescriptive requirements [10]. This includes Article 9 on protection and prevention which is relevant to the PQC migration [30]. Paragraph 1 of this Article stipulates that “financial entities shall continuously monitor and control the security and functioning of ICT systems and tools and shall minimise the impact of ICT risk on ICT systems through the deployment of appropriate ICT security tools, policies and procedures”. Paragraph 2 prescribes that

“Financial entities shall design, procure and implement ICT security policies, procedures, protocols and tools that aim to ensure the resilience, continuity and availability of ICT systems, in particular for those supporting critical or important functions, and to maintain high standards of availability, authenticity, integrity and confidentiality of data, whether at rest, in use or in transit.”

Pursuant to Article 15 of DORA, the European Commission adopted a delegated regulation on regulatory technical standards specifying ICT risk management tools, methods, processes, and policies to ensure further harmonisation thereof, which elaborates on many aspects of Article 9 of DORA. Recital 9 of this regulation acknowledges the threats arising from the development of quantum computers stating that

“Given the rapid technological developments in the field of cryptographic techniques, financial entities … should remain abreast of relevant developments in cryptanalysis and consider leading practices and standards. Financial entities … should hence follow a flexible approach, based on risk mitigation and monitoring, to deal with the dynamic landscape of cryptographic threats, including threats from quantum advancements” [18].

Moreover, this delegated regulation sets out detailed cryptographic guidance. For example, Article 6 obliges financial entities to develop and implement a policy on encryption and cryptographic controls. According to Article 6(3), such policy should include “criteria for the selection of cryptographic techniques and use practices, taking into account leading practices” and international, European or national standards. Furthermore, according to Article 6(4), such policy should include “provisions for updating or changing, where necessary, the cryptographic technology on the basis of developments in cryptanalysis. Those updates or changes shall ensure that the cryptographic technology remains resilient against cyber threats”. Financial entities that are not able to adhere to the above requirements “shall adopt mitigation and monitoring measures that ensure resilience against cyber threats”.

The DORA framework therefore imposes a dynamic approach to cryptographic management. It obliges financial entities to both keep up with leading practices in cryptographic technology as well as apply measures to remain resilient against cyber threats based on developments in cryptanalysis. This implies implementation of PQC, which, once standardised by dedicated international bodies or recommended by cybersecurity authorities, represents the state-of-the-art. In that, although it is not explicitly invoked, it can be argued that the DORA framework encompasses the precautionary approach.

## Conclusions

The financial sector is a critical domain where cyber threats represent systemic risks threatening wider financial stability. Quantum computers pose a new cybersecurity threat in that, once powerful enough, they are thought to be able to render most of the currently used encryption obsolete. To counter this risk, financial entities should adopt PQC as a precautionary measure.

The EU has recently adopted DORA—a comprehensive piece of legislation laying down stringent requirements in the field of financial cybersecurity. The DORA framework both recognises the systemic nature of cyber risks and acknowledges the threats arising from quantum advancements. This paper argues that its guidance on cryptographic management implies the obligation to adopt PQC and is congruent with the precautionary approach.
